# The Mysterious Universe of the TSH Receptor

**DOI:** 10.3389/fendo.2022.944715

**Published:** 2022-07-12

**Authors:** Inês Henriques Vieira, Dírcea Rodrigues, Isabel Paiva

**Affiliations:** Endocrinology, Diabetes and Metabolism Department of Coimbra Hospital and University Center, Coimbra, Portugal

**Keywords:** thyroid, thyroid stimulating hormone, TSH receptor autoantibodies, thyroid diseases, receptors, G-protein coupled receptors

## Abstract

The thyroid-stimulating hormone receptor (TSH-R) is predominantly expressed in the basolateral membrane of thyrocytes, where it stimulates almost every aspect of their metabolism. Several extrathyroidal locations of the receptor have been found including: the pituitary, the hypothalamus, and other areas of the central nervous system; the periorbital tissue; the skin; the kidney; the adrenal; the liver; the immune system cells; blood cells and vascular tissues; the adipose tissue; the cardiac and skeletal muscles, and the bone. Although the functionality of the receptor has been demonstrated in most of these tissues, its physiological importance is still a matter of debate. A contribution to several pathological processes is evident in some cases, as is the case of Grave’s disease in its multiple presentations. Conversely, in the context of other thyroid abnormalities, the contribution of the TSH-R and its ligand is still a matter of debate. This article reviews the several different sites of expression of the TSH-R and its potential role in both physiological and pathological processes.

## Introduction

The thyroid-stimulating hormone receptor (TSH-R), encoded by a gene located in 14q31 (1), belongs to the G protein-coupled receptor family. It has a large extracellular domain, seven transmembrane passages, and a small intracellular domain (2).

A single chained TSH-R with approximately 100 kDa has been described (3, 4), however, the holoreceptor is most frequently cleaved into two subunits, α and β, linked by disulfide bonds (5). Cleavage may be important in the induction of some of TSH-R signaling pathways (6). The membrane-spanning β subunit, with a molecular mass of ~30 kDa, is common to luteinizing hormone (LH), human chorionic gonadotropin (hCG) and follicle stimulating hormone (FSH). The α subunit, of ~50 kDa, and is TSH-specific, located in the extracellular region, and shed from the cell surface (2, 5).

The TSH-R couples to four subfamilies of G proteins (7): G_s_, inducing adenyl-cyclase activity and cyclic AMP production [the most common pathway (8, 9)]; G_q_/G_11_ activating phospholipase C; G_13_, inducing p44/42 mitogen-activated protein kinase (MAPK); G_i_ inhibiting adenyl-cyclase activity (7). The receptor is constitutionally active, however, TSH and TSHRAbs may enhance or, less frequently, block its signalling (5).

## TSH Receptor Expression

### In Thyroidal Tissue

The main site of expression of the TSH-R is the basolateral membrane of thyrocytes (10, 11). TSH-R activation stimulates iodine uptake, synthesis and secretion of thyroid hormones, and proliferation of thyroid follicular cells (9, 10, 12). In adult thyrocytes, TSH stimulation is paramount to maintaining follicular architecture and regulate the expression of thyroid-specific genes such as those coding thyroglobulin (Tg), thyroid peroxidase (TPO), and sodium/iodide symporter (NIS) (11).

TSH/TSH-R also seem to be relevant during the embryological development of the thyroid gland, as their expression was shown in embryonic stem cells (13). However, other factors are certainly involved at this stage (11).

### In Extrathyroidal Tissue

In the last decades, TSH-R expression has been found in several extrathyroidal tissues. In this section, data on the extrathyroidal sites of TSH-R expression and suggested, albeit generally unproved, physiological roles are explored.

TSH-releasing hormone (TRH) is produced in the hypothalamus mainly in the paraventricular nucleus and upregulates TSH production (14). The production of both TRH and TSH is under strict negative feedback control by the thyroid hormones (15). Follicle-stellate cells constitute ~10% of the anterior pituitary cells and form a network with each other and with endocrine cells (16). TSH-R expression was found in these cells and it has been hypothesized to be responsible for fine-tuning of the TSH levels, through an ultra-short negative feedback mechanism in pituitary thyrotrophic cells (16, 17). In the hypothalamus, activation of the TSH-R may be relevant for regulation of food intake (18) and influence seasonal reproductive patterns in some animals (19). TSH-R has also been demonstrated in other areas of the human brain such as the cortex, amygdala, cerebellum, cingulate gyrus and frontal, occipital and temporal lobes (20).

The presence of a functional TSH-R has been widely documented in the periorbital tissue (10, 21, 22), where it may be important in regulating the differentiation of orbital fibroblasts (23).

TSH-R is also expressed in the epidermis and hair follicles (24, 25), and the skin has been found to synthesize TSH under the control of TRH and thyroid hormones (23, 26). Treatment of organic cultures with TSH resulted in altered gene expression in hair follicles and stimulation of epidermis differentiation (25).

Thyroid hormones have been proposed to modulate renal development, morphology, and function (27). TSH-R expression was also demonstrated in the kidney and adrenal (28) and TSH stimulation was shown to increase cAMP production in human kidney cells (29).

In the ovary, the TSH-R has been found in granulosa cells (30) where its expression is increased by gonadotropins and decreased by estrogen (23). In murine models, the presence of TSH-R has been demonstrated in the testis and TSH has been shown to inhibit steroidogenesis (31). A role of TSH/TSH-R in the seasonal effects on gonadal growth in some animals has also been suggested (23).

TSH/TSH-R have been proposed to influence immune regulation (32). TSH-R expression was found in bone marrow hematopoietic cells, where TSH may regulate TNFα production (33). In thymocytes, TSH acting on the TSH-R was proposed to constitute an important growth factor influencing the development of T-cells (34). In white blood cells, TSH-R may be involved in recruitment, development and immunoregulation (23). This receptor has been found to be expressed on monocytes, dendritic cells, natural killer cells, T and B cells (32). Stimulation with recombinant human TSH has been shown to promote proliferation of natural killer cells, T and B cells (35). In dendritic cells, TSH stimulation was shown to lead to the production of pro-inflammatory cytokines and increased phagocytic activity (36).

Expression of the TSH-R in eritrocytes has been documented to influence Na^+^/K^+^-ATPase conformation (37). In blood vessels, TSH-R appears to contribute to the stimulation of angiogenesis and vascular smooth muscle proliferation (38).

TSH/TSH-R have been suggested to have bone protective properties (39). In human bone marrow-derived mesenchymal stem cells, TSH-R seems to be important for self-renewal, maintenance and differentiation (40). TSH was suggested to stimulate osteoblastic differentiation and to inhibit osteoclastogenesis (39). Nevertheless, sufficient data on the physiological effects of TSH on bone is conflicting (41). On the one hand, in human osteoblasts, TSH-R seems to have low expression and functionality (42). Also no influence of genetic variants influencing TSH concentration or TSH-R expression was found by van Vliet et al. (43). On the other hand, findings from van der Deure et al. and Kim et al. support an independent effect of TSH levels in improving bone mineral density (44).

Functional TSH-R was found in white adipose tissue in preadipocytes and differentiated adipocytes. It may have a role in preadipocyte behaviour and contribute to regulation of lipolysis in adipocytes (45, 46). In brown adipose tissue, a role of the TSH-R in thermogenesis has been suggested through induction of uncoupling protein-1 and deiodinase 2 (47, 48).

Expression of TSH-R has also been documented in hepatocytes (49), where stimulation with TSH may up-regulate cholesterol synthesis (50) and hepatic glucose production (51).

In the skeletal muscle, TSH appears to improve insulin sensitivity and increase insulin substrate-1 receptor expression (52). In the cardiac muscle, expression of a functional TSH-R has also been demonstrated (53, 54) and it was shown to influence cardiac electric properties (55).

As pleiotropic expression and myriad effects of the TSH-R are identified, it is becoming increasingly clear that it may play a part in several human diseases, which will be explored in the following sections of this text.

## TSH Receptor in Human Disease

### TSH Receptor Relevance in Thyroid Gland Diseases

Graves’ disease (GD) is the most common form of hyperthyroidism in countries without iodine deficiency (56, 57). Its physiopathology involves an autoimmune response with infiltration of specific T cells against the TSH-R, more commonly its thyroid-specific α-subunit (58). The fact that the α-subunit is shed may be important for the development of antibodies, but it is not sufficient, as it seems to occur in all humans (59). TSHRAbs share many of the actions of TSH on the TSH-R, and mostly lead to thyroid hyperplasia with upregulated production and secretion of thyroid hormone (5). However, TSH induces a more regulated response of thyroid specific genes, whereas stimulatory TSHRAbs persistently upregulate those genes (60). Conversely, some TSHRAbs may decrease TSH effects (blocking antibodies) or have a neutral effect on TSH binding and cAMP production (5).

Chronic autoimmune thyroiditis is an even more common autoimmune thyroid disease (61). Antibodies against thyroid peroxidase (anti-TPO) and thyroglobulin (anti-Tg) are usually present. However, TSHRAbs can be identified in 6,3-12% of HT patients and in 12-59% of atrophic thyroiditis patients. As some TSHRAbs have a blocking effect, they may contribute to hypothyroidism (5), or be associated with a fluctuating course between hyper and hypothyroidism (62).

Several somatic and germline mutations in the TSH-R have been identified and are listed on the TSH-R database (https://www.tsh-receptor-mutation-database.org/) (63).

Activating mutations of the TSH-R have been implicated in thyroid autonomy and hyperthyroidism (64, 65), these are usually located in exons 9 and 10 that encode the transmembrane domain (8). Inherited germline mutations are implicated in Familial Non-Autoimmune Autosomal Dominant Hyperthyroidism (OMIM 609152) (66), a rare, autosomal dominant, disease that courses with hyperthyroidism of varying severity and age of onset, and goiter. *De novo* germline mutations cause Persistent Sporadic Congenital Non-Autoimmune Hyperthyroidism, which usually presents precociously with significant hyperthyroidism, but with no familial history (8, 67).

Somatic activating mutations of the TSH-R are far more common and are involved in the pathogenesis of a substantial proportion of autonomous nodules and toxic multinodular goiter, the remaining being usually caused by somatic mutations in *GNAS* (68–70).

At the opposite pole, there are inactivating mutations that may occur in different parts of the receptor structure and cause resistance to TSH action (71). The clinical phenotype varies from compensated TSH resistance to congenital hypothyroidism with severe thyroid hypoplasia (23, 64). In most cases there seems to be a genotype-phenotype correlation with the former being associated with residual function of at least one allele, whereas hypothyroidism arises in the context of two non-functioning alleles (72).

Mutations that extend receptor specificity have also been described. TSH, FSH, LH and hCG and their receptors have evolved from a common ancestor. In normal conditions hCG can weakly stimulate the TSH-R leading to to lower TSH values in the first trimester of pregnancy (73). It has been proposed that mutations that reverse evolution may be associated with hyperemesis gravidarum, making TSH-R more sensitive to hCG action (23).

Finally, an important role of TSH-R in differentiated thyroid cancer (DTC) has also been proposed. Most, but not all, data suggests that TSH-R expression is maintained in tumor cells (74). TSH-R activation may have a pro-oncogenic and growth-promoting role (10). In murine models its expression was shown to be necessary for the initiation of the neoplastic process. TSH, acting through its receptor, has the potential of stimulating the growth of DTC (64). TSH has also been shown to promote vascular endothelial growth factor production thus contributing to angiogenesis (75, 76) to induce genomic instability (77) and to contribute to invasion and immune evasion in thyroid tumors (78). Conversely, the TSH-R seems to have an important role in maintaining differentiation of thyroid cancer cells, and in advanced and dedifferentiated thyroid cancer, a decrease in its expression has commonly been reported (79).

### TSH Receptor Relevance in Extrathyroidal Illness

Graves’ orbitopathy (GO) is present at GD presentation in ~26% of the cases, or, more rarely emerges during follow-up (80). It is an autoimmunity driven phenomena (81), causing orbital lymphocytic infiltration with a predominance of T lymphocytes, edema, and an increase in orbital connective tissue, adipose tissue and the extraocular muscles volume (82). The TSH-R is currently seen as the main autoantigen in ophthalmopathy and periorbital fibroblasts as the target of autoimmune attack (81). In patients with recent onset GO, TSHRAbs levels directly correlate with orbital disease activity (83) and may predict clinical course (84). Stimulation of TSH-R in orbital fibroblasts by TSHRAbs leads to activation of intracellular pathways, production of glycosaminoglycans and an increase in proliferation, adipogenesis and myofibrillogenesis (81). Indeed, enhanced TSH-R expression has been shown to be influenced by the autoimmune and inflammatory process (85) and to parallel with *de novo* adipogenesis (82). A role for the IGF-1 receptor has also been suggested and a crosstalk between IGF-1 receptor and the TSH-R is currently accepted as an important phenomenon in the pathophysiology of GO (86–90).

TSH-R has also been implicated in Graves’ dermatopathy. Similarly to what occurs with GO, this rare manifestation is associated with high titers of TSHRAbs, and characterized by a large amount of glycosaminoglycans dispersed in the reticular portion of the dermis. TSH-R immunoreactivity has been documented in the pretibium of patients with Graves’ dermatopathy (91).

The presence of TSH-R in thymocytes, may potentially explain the thymic hyperplasia seen in some patients with GD (34).

Hashimoto’s Encephalopathy, a rare aseptic form of encephalopathy, occurs in association with Hashimoto thyroiditis (92). Hypotheses proposed for its pathogenesis include: an immunopathological vasculitis; hormonal dysregulation; antibodies against antigens existent in the brain. The latter theory emphasizes the role of anti-thyroid antibodies such as TSHRAbs, anti-TPO and anti-Tg, since they are expressed in the brain (93). TSHRAbs might bind to TSH-R in cortical neurons and have a role in Hashimoto’s Encephalopathy (94). Homology between central nervous system antigens involved in Hashimoto’s Encephalopathy (such as alpha-enolase, dimethylargininase-I and aldehyde reductase-I) and thyroid antigens, including the TSH-R, has been found. This raises the possibility of cross-reactivity as an alternative pathophysiological mechanism (95).

Diffuse TSH-R expression in the brain may connect it with other neurological diseases. In the limbic system, abnormal interaction between anti-thyroid antibodies and the TSH-R may lead to neuronal inactivation/destruction and reduction of TSH-R expression, downregulating limbic-thyroid function, thus contributing to mood dysregulation and maniac-depressive disorders (20). Reduced TSH-R signaling may also be linked with declining cognitive function, as there is evidence suggesting an association between cognitive impairment and subclinical hyperthyroidism and in murine models, reduced TSH-R signaling was associated with impaired special learning and memory (96). TSHβ resistance has been associated with attention-deficit/hyperactivity disorder (ADHD) and TSH-R knockout in mice led to a ADHD phenotype (97). Conversely, both Alzheimer's disease and Down syndrome patients have greater expression of temporal and frontal lobe TSH-R, suggesting a potential role for TSH-R in neurogenerative disorders (20).

Thyroid disease is frequently accompanied by increased or decreased glomerular filtration rate or alterations in tubular transport (27), effects usually attributed to a direct action of thyroid hormones. As renal expression of TSH-R was documented, an influence of TSH itself has also been proposed (29). There are reports of nephritis due to thyroid antigen-antibody complexes in GD (98, 99). Despite these phenomena being generally attributed to circulating complexes, in light of the knowledge of TSH-R expression in the kidney, *in situ* antibody formation can also be considered (29).

Overt hypothyroidism has been associated with decreased fertility. For subclinical hypothyroidism this relationship is not clear, nevertheless TSH levels >4.0 mIU/L have been associated with adverse fertility outcomes (100). In a population with polycystic ovary syndrome undergoing *in vitro* fertilization, TSH levels in serum and in follicular fluid showed a negative correlation with oocyte maturation rate and fertilization (30). As such, one might wonder if TSH acting on the TSH-R in the granulosa cells, may contribute to the negative effects of hypothyroidism in fertility.

TSH acting on the testis may contribute to compromised secretion of androgens in hypothyroidism (31).

In murine models, absence of bone TSH-R expression was found to result in an osteoporotic phenotype. It is conceivable that the lack of TSH stimulation in thyrotoxicosis may contribute along with elevated thyroid hormones to increased bone loss in these patients (12, 101).

The presence of TSH-R on adipocytes has led some authors to question whether elevated TSH might contribute to the increased risk of obesity and cardiovascular disease associated with hypothyroidism (45). Overt hyperthyroidism has been associated with modest weight gain, however, for subclinical hypothyroidism a relationship with weight gain is not so clear. A positive correlation between TSH and body mass index has been found, although it’s been difficult to ascertain whether it is a contributing factor or a consequence of adiposity (102). There are some data to support a role of TSH-R signaling in the regulation of energy expenditure, thus contributing to weight variations associated with hyper and hypothyroidism (103). In murine models, a positive correlation between TSH-R expression and body mass index was found in diet-induced fat mice (104), and TSH-R knockout induced obesity resistance (105). It was proposed that TSH acting on the TSH-R on adipose tissue would promote triglyceride synthesis in adipocytes (105). A prior diagnosis of GD has been found to be a risk factor for a greater weight gain after treatment for hyperthyroidism (106) and it was hypothesized that TSHRAbs acting on adipose tissue might contribute to this effect (103).

Hypothyroidism is associated with abnormal cardiac repolarization and some data supports the possibility that increased stimulation of the TSH-R in cardiomyocytes may be a contributing factor (55, 107).

Since in hepatocytes TSH stimulation may increase both cholesterol and glucose synthesis, the presence of TSH-R in these cells may be one contributor to the worsening of cardiovascular risk factors associated with hypothyroidism (50, 51). Increased mitochondrial oxidative stress is associated with an incremental risk of conditions such as non-alcoholic hepatic liver disease. In murine models TSH signaling through its hepatic receptor has shown to upregulate oxidative stress (108).

Besides the above-mentioned role of the TSH-R in thyroid cancer, there are some data suggesting its expression in extrathyroidal malignancies such as melanoma (109), glioma/glioblastoma (110), lung (111), breast cancer (112), ovarian cancer (113) and hepatocellular carcinoma (114).

A summary of the locations of TSH-R and its potential influence on human disease is provided on **Table 1**.

**Table 1:** Locations of TSH-R and its potential influence on human disease.

Location	Proposed roles and Potential involvement in illness		References
**Thyroid**, basolateral membrane of thyrocytes (mRNA and protein)	• Stimulates almost every aspect of their metabolism • Possible role in embryonic development	• Graves’ Hyperthyrodism (TSHRABs) • Chronic autoimmune thyroiditis (blocking TSHRABs) • Familial non-autoimmune autosomal dominant hyperthyroidism, persistent sporadic congenital non-autoimmune hyperthyroidism, TSH resistance (germline mutations) • Autonomous nodules and toxic multinodular goiter (somatic mutations) • Thyroid cancer	(10, 11, 23, 58, 61, 64, 65, 71) .
**Pituitary** follicle-stellate cells (mRNA and protein)	• Ultra-short negative feedback mechanism	_	(16, 17)
**Hypothalamus** (mRNA and protein)	• Regulation of food intake • Influence in seasonal reproductive pattern	_	(18, 19)
**Other areas of the brain** (mRNA and protein)	_	• Hashimoto encephalopathy • Neurodegenerative disorders • Maniac depressive disorders	(20, 94, 96, 97)
**Periorbital tissue** (mRNA and protein)	• Differentiation of orbital fibroblasts	• Graves orbitopathy	(21, 23, 81, 82, 85)
**Epidermis and hair follicles** (mRNA and protein)	• Epidermis differentiation • Regulation of gene expression in hair follicles	• Graves dermatopathy	(24, 25)
**Ovary and Testis** (mRNA and protein)	• Regulation of sex steroid synthesis • Influence in seasonal reproductive pattern	• Negative effects of hypothyroidism in fertility. • Compromised androgen secretion in hypothyroidism.	(23, 30, 31)
**Immune system** (mRNA and protein – demonstrated in some cells)	• Regulation of recruitment, development and immunoregulatory functions • Regulation of TNFα production • Influence in the development of T-cells	• Thymic hyperplasia in Graves’s Disease • Immune dysregulation in cancer	(23, 33–36)
**Red blood cells** (protein)	• Na^+^/K^+^-ATPase conformation	_	(37)
**Blood vessels** (mRNA and protein)	• Stimulation of angiogenesis and vascular smooth muscle proliferation	• Increased angiogenesis in cancer	(38)
**Cardiomyocytes** (mRNA and protein)	• Influence cardiac electric properties	• Abnormal cardiac repolarization in hypothyroidism	(55, 107)
**Bone** (mRNA)	• Stimulation of osteoblastic differentiation • Inhibition of turnover and remodeling	• Decrease in bone mass in primary hyperthyroidism.	(39, 42)
**White adipose tissue** (mRNA and protein)	• Regulation of preadipocyte behaviour and lipolysis in adipocytes	• Obesity and increased cardiovascular risk (in hypothyroidism)	(45, 46)
**Brown adipose tissue** (mRNA)	• Stimulation of thermogenesis		(47, 48)
**Skeletal muscle** (mRNA and protein)	• Improvement of insulin sensitivity		(52)
**Kidney** and adrenal (mRNA and protein)	• Contribution to the influence on renal function of thyroid hormones	• Nephritis due to thyroid antigen-antibody complexes in GD (98, 99)	(28, 29, 98, 99)
**Liver** (mRNA and protein)	• Regulation of cholesterol synthesis and gluconeogenesis	• Contribution to hypercholesterolemia and altered glucose metabolism in the context of thyroid illness. • Increased oxidative stress.	(49–51, 108)

mRNA, messenger ribonucleic acid; TNFa, Tumor necrosis factor α; TSH, thyroid stimulating hormone; TSHRABs, antibodies against the TSH receptor.

## Concluding Remarks

The discovery of TSH-R expression in several organs changes the perspective of TSH action from a simple stimulator of thyroid gland function to a hormone with pleiotropic actions that may have an influence on the clinical picture of thyroid gland dysfunction and in several human diseases.

However, the physiological and pathophysiological roles are difficult to establish given that: it requires the ability to distinguish the consequences of TSH deficiency from those of thyroid hormones’ excess (reciprocal relationship), the receptor is frequently expressed at low levels in peripheral tissues, and there is the potential for local TSH production (12).

It is possible that the fact that TSH-R has a constitutive activation and a biphasic controlled response to TSH, may contribute to less overt manifestations of subclinical thyroid disorders.

Detailed examination of extrathyroidal manifestations of patients with germline TSH-R mutations rendered euthyroid may shed a light. New molecules with the function of TSH.R agonists, antagonists or inverse agonists have recently emerged, and can also assist in increasing our understanding on the extrathyroidal roles of the TSH-R. Due to the pleiotropic expression of the TSH-R, the importance of such knowledge may be reflected on several human diseases and even contribute to the creation of a new theranostic tool.

## Author Contributions

IV, DR, and IP were responsible for conceptualization and methodology; IV was responsible for literature review and preparation of the original draft; DR and IP supervised the manuscript creation and were responsible for its review and editing. All authors have read and agreed to the published version of the manuscript.

## Conflict of Interest

The authors declare that the research was conducted in the absence of any commercial or financial relationships that could be construed as a potential conflict of interest.

## Publisher’s Note

All claims expressed in this article are solely those of the authors and do not necessarily represent those of their affiliated organizations, or those of the publisher, the editors and the reviewers. Any product that may be evaluated in this article, or claim that may be made by its manufacturer, is not guaranteed or endorsed by the publisher.
